# Emotion regulation in patients with rheumatic diseases: validity and responsiveness of the Emotional Approach Coping Scale (EAC)

**DOI:** 10.1186/1471-2474-10-107

**Published:** 2009-09-03

**Authors:** Heidi A Zangi, Andrew Garratt, Kåre Birger Hagen, Annette L Stanton, Petter Mowinckel, Arnstein Finset

**Affiliations:** 1 National Resource Center for Rehabilitation in Rheumatology, Diakonhjemmet Hospital, Pb. 23 Vinderen, Oslo, Norway; 2 Department of Behavioural Science in Medicine, University of Oslo, Oslo, Norway; 3 Departments of Psychology and Psychiatry & Biobehavioral Sciences, University of California, Los Angeles, California, USA

## Abstract

**Background:**

Chronic rheumatic diseases are painful conditions which are not entirely controllable and can place high emotional demands on individuals. Increasing evidence has shown that emotion regulation in terms of actively processing and expressing disease-related emotions are likely to promote positive adjustment in patients with chronic diseases. The Emotional Approach Coping Scale (EAC) measures active attempts to acknowledge, understand, and express emotions. Although tested in other clinical samples, the EAC has not been validated for patients with rheumatic diseases. This study evaluated the data quality, internal consistency reliability, validity and responsiveness of the Norwegian version of the EAC for this group of patients.

**Methods:**

220 patients with different rheumatic diseases were included in a cross-sectional study in which data quality and internal consistency were assessed. Construct validity was assessed through comparisons with the Brief Approach/Avoidance Coping Questionnaire (BACQ) and the General Health Questionnaire (GHQ-20). Responsiveness was tested in a longitudinal pretest-posttest study of two different coping interventions, the Vitality Training Program (VTP) and a Self-Management Program (SMP).

**Results:**

The EAC had low levels of missing data. Results from principal component analysis supported two subscales, Emotional Expression and Emotional Processing, which had high Cronbach's alphas of 0.90 and 0.92, respectively. The EAC had correlations with approach-oriented items in the BACQ in the range 0.17-0.50. The EAC Expression scale had a significant negative correlation with the GHQ-20 of -0.13. As hypothesized, participation in the VTP significantly improved EAC scores, indicating responsiveness to change.

**Conclusion:**

The EAC is an acceptable and valid instrument for measuring emotional processing and expression in patients with rheumatic diseases. The EAC scales were responsive to change in an intervention designed to promote emotion regulation. The instrument has not yet been tested for test-retest reliability, which is recommended in future studies.

## Background

Chronic rheumatic diseases often have an important impact on physical, as well as psychological and social aspects of patients' lives. Such long-term stressors that have uncontrollable elements can place great emotional demands on the individual. Research has documented a high degree of depression, anxiety and psychological distress in patients with rheumatoid arthritis (RA) and other rheumatic diseases [1-6].

There are individual differences in how patients cope with various symptoms and adjust to the burden of the disease. In early stages of the disease emotional distress is associated with levels of pain and fatigue, functional status, disease impact on daily life, life events and perceived social support [1,7]. The effect of disease-related factors on psychological distress seems to decrease [3,8] and personality characteristics and individual coping resources appear to become more important predictors over time [7,9]. Various self-management interventions have been developed to improve patients' ability to cope with the complexity of symptoms related to their rheumatic disease [10-12]. There is a growing consensus that emotion regulation, in terms of acknowledging and dealing with negative emotions associated with chronic illness, can contribute to adjustment [13,14]. However, few interventions explicitly address patients' emotional response to their disease [9,15].

The Vitality Training Program (VTP) is a Norwegian group intervention [16], which has a special focus on awareness of and reflection upon one's own emotions, thoughts and bodily experiences. It has produced a reduction in psychological distress and increased emotional well-being in a randomized, controlled trial in persons with chronic musculoskeletal pain [17] and in a one-year follow-up of a non-controlled study for patients with inflammatory rheumatic diseases [18]. The results shall be further evaluated in a randomized, controlled trial. However, following a search of the literature, no acceptable instrument to measure intervention-related change in patients' coping strategies related to emotional awareness was found.

The coping literature makes a distinction between problem-focused and emotion-focused coping strategies [19-21]. Problem-focused coping includes direct efforts to alter the demands on the person, whereas emotion-focused coping includes efforts to regulate emotions associated with stressful situations. Research based on earlier published coping scales has found that use of emotion-focused coping strategies is often associated with psychological distress and maladjustment [13,20,22]. However, there is increasing research on the adaptive nature of acknowledging, processing, and expressing one's emotions. An ability to approach one's and others' emotions is seen as crucial to healthy intra- and interpersonal functioning [13-15,23]. This view has also received empirical support through studies on emotional disclosure interventions [24-29].

Major problems have been identified in the existing conceptualization and measurement of emotion-focused coping [13,20,22]. Diverse coping methods, such as approach-oriented (e.g. *seeking social support*) and avoidance-oriented (e.g. *denial*) strategies, have been assessed with emotion-focused coping items, some of which are inversely correlated. Emotion-focused coping scales have also included items related to emotional distress and negative emotions (e.g. *angry, upset, blame myself*). Hence, these earlier measures of emotion-focused coping were confounded by aspects of psychological health. Stanton et al [22] developed a construct of emotional approach coping which does not include distress-related items. Based on this construct, the Emotional Approach Coping Scale (EAC) is comprised of two factors [22]: Emotional Processing, which includes active attempts to acknowledge, explore meanings and come to an understanding of one's emotions; and Emotional Expression, which includes active verbal and/or nonverbal attempts to communicate or symbolize one's emotional experience. The EAC has been tested in clinical samples [13], including patients with chronic myofascial pain [29]. These studies show that emotional approach coping is inversely related to psychological distress and can predict better adjustment and less pain and depression over time [13,22,29].

The aim of this study was to evaluate the Norwegian version of the EAC in patients with rheumatic diseases. More specifically, the instrument was tested for data quality, internal consistency reliability and construct validity in a cross-sectional study. Responsiveness to change was assessed using longitudinal data from a pretest-posttest study that included patients recruited from two different coping interventions, the VTP and an inpatient Self-Management Program (SMP).

## Methods

### Data collection

Recruitment of three different groups of patients took place in 2007 and all received self-completed questionnaires. For the cross-sectional study, 118 consecutive patients (group 1) who had a confirmed rheumatological diagnosis and who attended regular consultations at rheumatology inpatient, outpatient or day hospital clinics, were recruited. Questionnaires were given to patients by rheumatology nurses at a single data collection point. For the pretest-posttest study, 49 patients (group 2) attending four VTP groups and 103 patients (group 3) attending five diagnosis-specific SMP courses were recruited. Group 2 were given questionnaires by facilitators immediately before the first VTP session and were mailed questionnaires after the last session. Group 3 were given the questionnaires by a nurse at the beginning of the first day of the SMP and at the end of the last day of the course. Baseline data from group 2 and 3 and all data from group 1 were used in the cross-sectional study.

Inclusion criteria were adults with confirmed rheumatological diagnosis in the consecutive sample (group 1) and confirmed rheumatological diagnosis of at least six months duration and age over 20 years in the two coping interventions (group 2 and 3). All patients received written and oral information regarding the study, and informed consent was obtained from all patients before entering the study. Group 1 patients were free to either fill out the questionnaire or not; questionnaires were sent back anonymously. This survey was regarded as a component of quality assurance and hence ethical approval was not required. The pretest-posttest study samples participated in two evaluation studies, which both were approved by the Regional Committee for Medical Ethics.

### Interventions

The VTP aims at helping patients to become more aware of their internal and external resources in order to cope with their current life situation. Processing, acknowledging, and expressing one's emotions are central elements. Through participation-based teaching and counselling methods, as well as awareness and relaxation training, patients learn to accept their thoughts, feelings and bodily experiences as they are, and respond to their symptoms, other people, and situations in more adaptive ways. The VTP lasts for approximately 4 months, involving 10 sessions of process-oriented group learning for 8 to 12 patients with different rheumatic diseases. Each group is facilitated by two health professionals (nurses, physiotherapists, occupational therapists or social workers), who have completed a one-year postgraduate program in the VTP. The one-week inpatient Self-Management Program (SMP) is taught in disease specific groups for 16 patients, consisting of consultation with a rheumatologist, multidisciplinary disease specific teaching, physical exercises and sharing experiences in small groups. The aim of this intervention is to strengthen the patients' ability to manage symptoms, treatment, and physical and psychosocial life style changes inherent in living with a rheumatic disease.

### Measures

The EAC comprises 16 items that form two subscales; Emotional Processing (8 items) and Emotional Expression (8 items). The items have a 4-point scale of "I don't do this at all", "I do this a little bit", "I do this a medium amount" and "I do this a lot". The mean item score is calculated for each subscale. The two subscales have satisfactory internal consistency and test-retest reliability in samples of undergraduate students [22].

The original US version of the EAC underwent a forward and backward translation [30]. Two Norwegians proficient in English independently translated the questionnaire into Norwegian. One licensed translator, and a person who was proficient in English carried out the backward translation independently. The forward and backward translations were discussed by the translators, a psychologist and the research group. Discrepancies between the various versions were resolved by consensus in order to achieve conceptual equivalence between the Norwegian and original US versions of the EAC. The consensus version of the back-translation was mailed to the original author (ALS) who confirmed semantic equivalence. In the current study, participants were instructed to complete the items with reference to how they usually respond to emotions related to their chronic rheumatic disease.

The Brief Approach/Avoidance Coping Questionnaire (BACQ) comprises 12 items designed to measure a general concept of approach-versus avoidance-oriented coping. Items have a 5-point Likert scale from "strongly disagree" to "strongly agree". The BACQ had satisfactory psychometric properties in a Norwegian population of primary care patients [31].

The General Health Questionnaire (GHQ-20) comprises 20 items and measures several aspects of psychological distress during the previous 2 weeks [32,33]. Items have a 4-point scale of "not at all", "no more than usual", "rather more than usual" and "much more than usual". The total score ranges from (0) no distress at all to (60) severe distress. The GHQ has been shown to be valid and reliable across cultures [34] and the GHQ-20 has been widely used in Norwegian studies [35,36].

### Statistical analyses

Levels of missing data were assessed for the EAC items and scales. Evidence for the existence of the two EAC subscales was assessed by principal component analyses (PCA) with varimax rotation [37]. Components with eigenvalues greater than one were considered potentially important.

#### Internal consistency

Internal consistency was assessed by item-total correlations and Cronbach's alpha. For a scale to be sufficiently reliable for use in groups of patients, an alpha value of 0.70 is considered acceptable [38,39].

#### Construct validity

Construct validity was assessed by comparing the EAC scores with the BACQ and GHQ-20 with Pearson's correlation coefficient [39-41]. We hypothesised that the EAC scores would be positively related to approach-oriented items in the BACQ. EAC subscales and the BACQ measure distinct aspects of coping, and hence correlations with the BACQ were not expected to be high, but of a low to moderate level (0.3 - 0.6). Because the EAC scales were designed to be unconfounded by distress-laden content [22], we expected the correlations with GHQ-20 to be negative and of a low magnitude under 0.3.

#### Responsiveness to change

Responsiveness refers to an instrument's ability to measure change over time [39,42,43]. This was assessed in the VTP and the SMP samples by the standardized response mean (SRM). SRMs were calculated by dividing the mean change scores by the standard deviation (SD) of the change scores. Effective processing and expression of emotions are more central elements in the VTP than in the SMP, and hence it was hypothesised that relative to SMP participants, the VTP participants would have a larger increase in the EAC scores.

All analyses were performed with SPSS, version 14.0 (SPSS Inc., Chicago, IL, USA).

## Results

### Data collection

A total of 220 patients were included in the cross-sectional study; 118 in group 1, 66 (64.1%) in group 2 and 36 (73.5%) in group 3 (table 1). Patients had a mean age of 50.3 (SD 12.63), 165 (75.0%) were women and following diagnoses were reported: rheumatoid arthritis (58), ankylosing spondylitis (38), psoriatic arthritis (32), fibromyalgia (31), osteoarthritis (25), connective tissue diseases (11), juvenile rheumatoid arthritis (4) and others (18). Group 1 patients attending the rheumatology clinic were more likely to be men and had significantly longer disease duration than the two others. Group 3 patients attending the VTP, were significantly younger than the two other groups. No significant differences were found for other variables assessed at baseline (table 1).

**Table 1:** Baseline characteristics of patients (n = 220)

	**Group 1**	**Group 2**	**Group 3**
	**Rheumatology**	**Vitality Training**	**Self-Management**

	**clinics (n = 118)**	**Program (n = 36)**	**Program (n = 66)**

			

Age, mean (range)	49.9 (17 - 78)	45.9 (29 - 71)*	53.3 (25 - 86)

Female, n(%)	71 (60.7)**	34 (94.4)	60 (90.9)

Disease duration, mean (range)	10.0 (0 - 47)***	5.0 (1 - 45)	5.0 (0.5 - 32)

*Diagnosis, n (%):*			

rheumatoid arthritis	48 (41.7)	10 (27.8)	0

ankylosing spondylitis	22 (19.1)	6 (16.7)	19 (15.2)

psoriatic arthritis	20 (17.4)	5 (13.9)	7 (10.6)

fibromyalgia	2 (1.7)	8 (22.2)	21 (31.8)

connective tissue disease	5 (4.3)	0	6 (9.1)

osteoarthritis	1 (0.9)	2 (5.6)	22 (33.3)

juvenile arthritis	3 (2.6)	1 (2.8)	0

others	14 (12.2)	4 (11.1)	0

			

*EACscales:*			

Emotional Processing	2.78 (0.6)	2.96 (0.6)	2.83 (0.7)

Emotional Expression	2.68 (0.6)	2.60 (0.7)	2.76 (0.6)

BACQ	3.28 (0.4)	3.25 (0.5)	3.15 (0.4)

GHQ - 20	22.01 (9.8)	24.06 (7.8)	22.48 (9.6)

Emotional Processing: 1 = low processing, 4 = high processing

Emotional Expression: 1 = low expression, 4 = high expression

BACQ = Brief Approach/Avoidance Coping Questionnaire: 1 = low approach, 4 = high approach

GHQ - 20 = General Health Questionnaire: 0 = no distress, 60 = high distress

Values are means (SD) for continues variables and frequencies (percentages within sample) for categorical values.

Disease duration = median years since diagnosis.

* significant lower mean than the two other samples (*p *= 0.027)

** significant lower proportion than the two other samples (*p *< 0.001)

*** significant higher median than the two other samples (*p *< 0.001)

### Statistical analyis

Overall, patients were able to complete the EAC questionnaire without help. There were few missing values, and these ranged from 1.9% to 5.7% for items relating to Emotional Expression and Emotional Processing, respectively. Mean item scores (SD) ranged from 2.51 (0.89) to 3.32 (0.66) (table 2).

**Table 2:** Descriptive statistics, component loadings and internal consistency of the EAC (n = 220)

**Frequency %**								
**Scale/item**	**Missing (%)**	**Mean (SD)**	**Not at all**	**A little**	**Moderately**	**A lot**	**Component loading^a^**	**Cronbach's α/item-total correlation**
Emotional processing^b^		2.84 (0.63)	-	-	-	-	-	0.90
Take time to figure out	1 (0.5)	2.79 (0.77)	8 (3.6)	73 (33.2)	100 (45.5)	38 (17.3)	0.71	0.88
Delve into feelings	0	2.62 (0.83)	14 (6.4)	92 (41.8)	79 (35.9)	35 (15.9)	0.81	0.88
Validity/importance	3 (1.4)	3.28 (0.69)	6 (2.7)	17 (7.7)	105 (47.7)	89 (40.5)	0.58	0.90
Acknowledge emotions	5 (2.4)	3.32 (0.66)	0	25 (11.4)	96 (43.6)	94 (42.7)	0.46	0.90
Work on understanding	0	2.88 (0.84)	11 (5.0)	64 (29.1)	87 (39.5)	58 (26.4)	0.84	0.88
Explore emotions	2 (0.9)	2.51 (0.89)	30 (13.6)	85 (38.6)	72 (32.7)	31 (14.1)	0.80	0.88
Find way to understand	1 (0.5)	2.69 (0.84)	15 (6.8)	82 (37.3)	84 (38.2)	38 (17.3)	0.80	0.88
Look closely at reasons	0	2.72 (0.91)	20 (9.1)	72 (32.7)	79 (35.9)	49 (22.3)	0.85	0.88
Emotional expression^b^		2.71 (0.63)	-	-	-	-	-	0.92
Take time to express	0	2.62 (0.76)	10 (4.5)	89 (40.5)	94 (42.7)	27 (12.3)	0.62	0.91
Let feelings come out	0	2.57 (0.84)	21 (9.5)	84 (38.2)	87 (39.5)	28 (12.7)	0.79	0.91
Allow myself to express	0	2.75 (0.81)	14 (6.4)	62 (28.2)	110 (50.0)	34 (15.5)	0.85	0.90
Feel free to express	0	2.92 (0.84)	15 (6.8)	45 (20.5)	109 (49.5)	51 (23.2)	0.75	0.91
Express feelings I have	1 (0.5)	2.77 (0.74)	8 (3.6)	66 (30.0)	113 (51.4)	32 (14.5)	0.86	0.90
Find way to express	2 (0.9)	2.80 (0.72)	8 (3.6)	58 (26.4)	122 (55.5)	30 (13.6)	0.62	0.92
Let feelings out	0	2.65 (0.82)	16 (7.3)	85 (38.6)	88 (40.0)	31 (14.1)	0.87	0.90
Get feelings out	1 (0.5)	2.67 (0.77)	13 (5.9)	80 (36.4)	100 (45.5)	26 (11.8)	0.82	0.90

^a ^Emotional expression and emotional processing had eigenvalues of 7.25 and 2.79 respectively

^b ^The two EAC scales are scored from 1-4 where 4 reflects the most frequent use of EAC

Principal component analysis yielded two components with eigenvalues 7.25 and 2.79; a third weak component had an eigenvalue of 0.97. The first two components accounted for 62.7% of the total variance. Both components had high loadings, the majority (14/16) being over 0.6 and clearly reflecting the two hypothesized domains of Emotional Expression and Emotional Processing.

#### Internal consistency

Item-total correlations all exceeded 0.45. Both the EAC Expression and Processing scales met the Cronbach's alpha criterion of 0.70.

There were very little missing data at the scale level. Whilst item scores were generally skewed towards positive levels of coping, the domain scores were more normally distributed with means of 2.84 (0.63) and 2.71 (0.63) for Emotional Processing and Emotional Expression, respectively (table 2). End effects were low; 3 (1.36%); and 0 patients scored at the floor and 10 (4.55%) and 14 (6.36%) scored at the ceiling for Emotional Expression and Emotional Processing, respectively.

#### Construct validity

As hypothesised the EAC scales had low to moderate correlations with the approach-oriented items in the BACQ. The EAC Expression scale had significant moderate correlations with the BACQ items "I say so if I am angry or sad" and "I like to talk to a few chosen people when things get too much for me", but had only weak correlations with the other approach-oriented items. The EAC Processing scale had a significant moderate correlation with the item "I like to talk to a few chosen people when things get too much for me" and was only weakly correlated with the other approach-oriented items (table 3). There were no significant correlations between the EAC scales and the avoidance-oriented items in BACQ. The EAC Expression scale had a low, but significant, negative correlation with the GHQ-20, such that higher emotional expression was associated with lower distress, supporting the hypothesis that the EAC scales are unconfounded of distress-laden content. The correlation between the EAC Processing scale and the GHQ-20 was very low and not significant.

**Table 3:** Correlation between the EAC scores, BACQ and GHQ-20 (n = 220)

	**EAC processing**	**EAC expression**
EAC processing	-	0.48**

EAC expression	0.48**	-

BACQ total	0.29**	0.40**

1 I say so if I am angry or sad	0.18*	0.50**

2 I like to talk to a few chosen people when things get too much for me	0.39**	0.44**

4 I make an active effort to find a solution to my problems	0.26**	0.17*

8 I think something positive could come out of my complaints/problems	0.24**	0.22**

9 I firmly will believe that my problems will decrease	0.08	0.13*

GHQ-20	- 0.01	-0.21*

* *p *< 0.05, ** *p *< 0.01

#### Responsiveness to change

Twenty-six (72%) of the included patients in group 2 completed a questionnaire after the VTP. All included group 3 patients attending the SMP completed a questionnaire after the program. As hypothesised the EAC scores increased more in the VTP sample than in the SMP sample. In the VTP sample, there were significant increases in both EAC Processing and EAC Expression. This group also showed a significant reduction in GHQ-20 scores. The SRMs were between 0.40 and 0.73. There were no significant changes in the EAC scales in the SMP sample at follow-up, but a significant reduction was found in the GHQ-20 scores (table 4).

**Table 4:** Responsiveness of the EAC, BACQ and GHQ-20 in the VTP^§ ^(n = 26) and the SMP^§ ^(n = 66)

	**Baseline**	**Follow-up**	**Change scores**	**SRM**
	**mean (SD)**	**mean (SD)**	**mean (SD)**	

EAC processing				

VTP (4 months)	3.12 (0.60)	3.39 (0.64)	- 0.27 (0.58)*	0.47

SMP (1 week)	2.83 (0.66)	2.93 (0.66)	- 0.10 (0.52)	0.19

EAC expression				

VTP (4 months)	2.69 (0.64)	3.06 (0.56)	- 0.37 (0.53)**	0.70

SMP (1 week)	2.76 (0.63)	2.82 (0.57)	- 0.06 (0.45)	0.13

BACQ total				

VTP (4 months)	3.41 (0.42)	3.55 (0.46)	- 0.14 (0.35)	0.40

SMP (1 week)	3.14 (0.44)	3.22 (0.45)	- 0.08 (0.36)	0.22

GHQ-20				

VTP (4 months)	24.88 (9.40)	16.23 (9.33)	8.65 (11.80)**	0.73

SMP (1 week)	22.94 (9.96)	17.55 (8.89)	5.39 (7.15)**	0.75

^§ ^VTP = the Vitality Training Program, SMP = the Self Mangement Program

* P < 0.05, ** *P *< 0.001, Values refer to statistical differences in mean scores between T1 and T2

SD = Standard deviation

SRM = Standardized Response Mean

## Discussion

This study has translated the EAC to Norwegian following established guidelines for forward-backwards translation [30]. The Norwegian version of the EAC was found to be acceptable to patients with rheumatic diseases and had low levels of missing data. The results of PCA show that the two scales defined by the instrument's authors are supported empirically. The EAC scales also have good evidence for internal consistency and construct validity. As hypothesized, the EAC scales were responsive to change in an intervention designed to promote effective emotion regulation (VTP), but not in an intervention with less focus on managing emotions (SMP).

The items "I realize that my feelings are valid and important" and "I acknowledge my emotions" had the lowest item-total correlations. The content of these two items may be interpreted as reflecting the value of one's emotions. They loaded satisfactorily on the emotional processing factor, but might as well have contributed to a third factor. Looking at the skewed distributions of these two items, we also found a ceiling effect, which may reflect a high threshold for saying that one's emotions are not valid and acknowledged. There were some missing values for these items, and hence they may have been more difficult to interpret for some patients.

Both EAC scales have high internal consistency which follow previous findings [22,29]. The high Cronbach's alpha for the EAC Expression scale (0.92) may indicate that some of the items in this scale are unnecessary. An earlier version of the EAC comprised two four-item scales [22] which were expanded to eight-item scales. The longer version was found to have slightly higher reliability, which was the reason for choosing the longer-form version for this cross-cultural adaptation. If the EAC is to be used with groups of patients, then it might be argued that the four-item scales will suffice. However, if the instrument is to be used in clinical practice for assessing individual patients then the longer-form is recommended since it meets the minimum criterion of a Cronbach's alpha of 0.90 [44].

Both EAC scales were related to the approach-oriented items in the BACQ and uncorrelated with the avoidance-oriented items, supporting construct validity. As expected, the correlations were strongest between EAC Expression and the expressive BACQ items. The inverse correlation between EAC Expression and the GHQ-20 scores supports the hypothesis that EAC Expression is related to positive adjustment, rather than dysfunction. From the negative correlation it may be concluded that the concept of coping through expressing one's emotions is distinct from psychological distress. Taken together, these findings correspond with previous studies that concluded that emotional approach coping is conceptually different from passive, avoidance-oriented coping and from other emotion-focused coping strategies related to distress [22,29].

The findings supported the hypothesis that patients with rheumatic diseases participating in the VTP would increase their EAC, whereas participants in the SMP would not. The two interventions are different in both length and content. In the SMP, active problem-focused coping strategies are emphasized. Even though emotional distress is a topic in the program, the participants are not invited to explore and understand their own emotional coping strategies to the same degree as in the VTP. Therefore it was not expected that this one-week program would change emotion-focused coping strategies. In contrast, the VTP is a four-month process-oriented program with a special focus on emotions, and hence a change in emotion-focused coping strategies was expected. Both EAC scale scores improved significantly after the VTP. The smaller, significant change in EAC Processing may be due to the relatively high score at baseline, making it difficult to detect change. There were also a small number of patients in the VTP sample so the results should be interpreted with some caution. Few studies have examined EAC as an outcome or moderator of effects of emotion regulation interventions [22,28,45-48]. Findings in this study indicate that the EAC may be used as an outcome measure in interventions for patients with rheumatic diseases where emotional approach coping is targeted.

A limitation in this study is that the EAC was compared with only two other measures to assess construct validity and comparisons with other scales measuring approach/avoidance-oriented coping and active/passive coping are recommended. Another limitation is that the instrument has not yet been tested for test-retest reliability, which is recommended in future studies. Test-retest data would also have allowed the calculation of the responsiveness statistic, which takes account of score variation in patients who are stable [49]. Receiver operating characteristic (ROC) curves [50] is a further method of assessing responsiveness but the absence of a criterion relating to whether patients had an important improvement in coping limited the scope for ROC analysis in this study. The identification of patients that have had an important change in their coping based on clinical or patient judgments can also facilitate the interpretability of EAC subscale scores [38]. The short follow-up period in the pretest-posttest study is a further study limitation, which limits the conclusions relating to responsiveness.

## Conclusion

The results of this study show that the EAC has evidence for data quality, internal consistency and validity, but has yet to be assessed for test-retest reliability, which is recommended in future studies. The study has also shown that participation in the VTP improves emotion-focused coping strategies as measured by the EAC in patients with various rheumatic diseases, showing that the EAC scales are responsive to change. Further studies based on larger samples with a longer follow-up period that include other important psychological and health-related outcome measures are recommended.

## Competing interests

The authors declare that they have no competing interests.

## Authors' contributions

HAZ, KBH and AF conceived the study; ALS developed the original instrument; HAZ coordinated the data collection; PM, AG, KBH and HAZ performed the statistical analyses; HAZ, AG, KBH, AF and ALS drafted the manuscript. All authors read and approved the final manuscript.

## Pre-publication history

The pre-publication history for this paper can be accessed here:



## References

[B1] Smedstad LM, Moum T, Vaglum P, Kvien TK (1996). The impact of early rheumatoid arthritis on psychological distress. A comparison between 238 patients with RA and 116 matched controls. Scand J Rheumatol.

[B2] Danoff-Burg S, Revenson TA, Trudeau KJ, Paget SA (2004). Unmitigated communion, social constraints, and psychological distress among women with rheumatoid arthritis. J Pers.

[B3] Strating MM, Suurmeijer TP, van Schuur WH (2006). Disability, social support, and distress in rheumatoid arthritis: results from a thirteen-year prospective study. Arthritis Rheum.

[B4] Kozora E, Ellison MC, Waxmonsky JA, Wamboldt FS, Patterson TL (2005). Major life stress, coping styles, and social support in relation to psychological distress in patients with systemic lupus erythematosus. Lupus.

[B5] Dickens C, McGowan L, Clark-Carter D, Creed F (2002). Depression in rheumatoid arthritis: a systematic review of the literature with meta-analysis. Psychosom Med.

[B6] VanDyke MM, Parker JC, Smarr KL, Hewett JE, Johnson GE, Slaughter JR, Walker SE (2004). Anxiety in rheumatoid arthritis. Arthritis Rheum.

[B7] Evers AW, Kraaimaat FW, Geenen R, Bijlsma JW (1997). Determinants of psychological distress and its course in the first year after diagnosis in rheumatoid arthritis patients. J Behav Med.

[B8] Smedstad LM, Vaglum P, Moum T, Kvien TK (1997). The relationship between psychological distress and traditional clinical variables: a 2 year prospective study of 216 patients with early rheumatoid arthritis. Br J Rheumatol.

[B9] Evers AW, Kraaimaat FW, Geenen R, Jacobs JW, Bijlsma JW (2002). Longterm predictors of anxiety and depressed mood in early rheumatoid arthritis: a 3 and 5 year followup. J Rheumatol.

[B10] Koehn CL, Esdaile JM (2008). Patient education and self-management of musculoskeletal diseases. Best Pract Res Clin Rheumatol.

[B11] Newman S, Steed L, Mulligan K (2004). Self-management interventions for chronic illness. Lancet.

[B12] Warsi A, Wang PS, LaValley MP, Avorn J, Solomon DH (2004). Self-management education programs in chronic disease: a systematic review and methodological critique of the literature. Arch Intern Med.

[B13] Austenfeld JL, Stanton AL (2004). Coping through emotional approach: a new look at emotion, coping, and health-related outcomes. J Pers.

[B14] de Ridder D, Geenen R, Kuijer R, van Middendorp H (2008). Psychological adjustment to chronic disease. Lancet.

[B15] Keefe FJ, Lumley M, Anderson T, Lynch T, Studts JL, Carson KL (2001). Pain and emotion: new research directions. J Clin Psychol.

[B16] Steen E, Haugli L (2000). The body has a history: an educational intervention programme for people with generalised chronic musculoskeletal pain. Patient Educ Couns.

[B17] Haugli L, Steen E, Lærum E, Nygård R, Finset A (2003). Psychological distress and employment status in patients with chronic musculoskeletal pain: Results from a group learning program based on personal construct theory. Psychology, Health & Medicine.

[B18] Zangi HA, Finset A, Steen E, Mowinkel P, Hagen KB (2009). The effects of a vitality training programme on psychological distress in patients with inflammatory rheumatic diseases and fibromyalgia: a 1-year follow-up. Scand J Rheumatol.

[B19] Carver CS, Scheier MF, Weintraub JK (1989). Assessing coping strategies: a theoretically based approach. J Pers Soc Psychol.

[B20] Stanton AL, Danoff-Burg S, Cameron CL, Ellis AP (1994). Coping through emotional approach: problems of conceptualization and confounding. J Pers Soc Psychol.

[B21] Lazarus RS, Folkman S (1984). Stress Appraisal and Coping.

[B22] Stanton AL, Kirk SB, Cameron CL, Danoff-Burg S (2000). Coping through emotional approach: scale construction and validation. J Pers Soc Psychol.

[B23] Levenson RW, Ekman P, Davidson RJ (1994). Human Emotions: A Functional View. The Nature of Emotions Fundamental Questions.

[B24] Pennebaker JW (2000). Telling stories: the health benefits of narrative. Lit Med.

[B25] Smyth JM, Stone AA, Hurewitz A, Kaell A (1999). Effects of writing about stressful experiences on symptom reduction in patients with asthma or rheumatoid arthritis: a randomized trial. JAMA.

[B26] Kelley JE, Lumley MA, Leisen JC (1997). Health effects of emotional disclosure in rheumatoid arthritis patients. Health Psychol.

[B27] Stanton AL, Danoff-Burg S, Sworowski LA, Collins CA, Branstetter AD, Rodriguez-Hanley A (2002). Randomized, controlled trial of written emotional expression and benefit finding in breast cancer patients. J Clin Oncol.

[B28] Austenfeld JL, Paolo AM, Stanton AL (2006). Effects of writing about emotions versus goals on psychological and physical health among third-year medical students. J Pers.

[B29] Smith JA, Lumley MA, Longo DJ (2002). Contrasting emotional approach coping with passive coping for chronic myofascial pain. Ann Behav Med.

[B30] Beaton DE, Bombardier C, Guillemin F, Ferraz MB (2000). Guidelines for the process of cross-cultural adaptation of self-report measures. Spine.

[B31] Finset A, Steine S, Haugli L, Steen E, Lærum E (2002). The Brief Approach/Avoidance Coping Questionnaire: development and validation. Psychology, Health & Medicine.

[B32] Goldberg DP, Williams P (1988). A Users Guide to the General Health Questionnaire GHQ.

[B33] Malt UF, Mogstad TE, Refnin IB (1989). [Goldberg's General Health Questionnaire]. Tidsskr Nor Laegeforen.

[B34] Furukawa T, Goldberg DP (1999). Cultural invariance of likelihood ratios for the General Health Questionnaire. Lancet.

[B35] Malt UF (1989). The validity of the General Health Questionnaire in a sample of accidentally injured adults. Acta Psychiatr Scand Suppl.

[B36] Grotle M, Hagen KB, Natvig B, Dahl FA, Kvien TK (2008). Prevalence and burden of osteoarthritis: results from a population survey in Norway. J Rheumatol.

[B37] Jollife IT (2002). Principal Component Analysis.

[B38] Fitzpatrick R, Davey C, Buxton MJ, Jones DR (1998). Evaluating patient-based outcome measures for use in clinical trials. Health Technol Assess.

[B39] Streiner DL, Norman GR (2003). Health Measurement Scales - a practical guide to their development and use.

[B40] Polit DF, Hungler PB (2007). Nursing Research Principles and Methods.

[B41] Guyatt GH, Feeny DH, Patrick DL (1993). Measuring health-related quality of life. Ann Intern Med.

[B42] Wiebe S, Guyatt G, Weaver B, Matijevic S, Sidwell C (2003). Comparative responsiveness of generic and specific quality-of-life instruments. J Clin Epidemiol.

[B43] Middel B, van Sonderen E (2002). Statistical significant change versus relevant or important change in (quasi) experimental design: some conceptual and methodological problems in estimating magnitude of intervention-related change in health services research. Int J Integr Care.

[B44] Nunnally JC, Bernstein IH (1994). Psychometric theory.

[B45] Austenfeld JL, Stanton AL (2008). Writing about emotions versus goals: effects on hostility and medical care utilization moderated by emotional approach coping processes. Br J Health Psychol.

[B46] Antoni MH, Lehman JM, Kilbourn KM, Boyers AE, Culver JL, Alferi SM (2001). Cognitive-behavioral stress management intervention decreases the prevalence of depression and enhances benefit finding among women under treatment for early-stage breast cancer. Health Psychol.

[B47] Manne S, Ostroff JS, Winkel G (2007). Social-cognitive processes as moderators of a couple-focused group intervention for women with early stage breast cancer. Health Psychol.

[B48] Antoni MH, Carver CS, Lechner SC, Park CL, Lechner SC, Antoni MH, Stanton AL (2009). Enhancing positive adaptation: Example intervention during treatment for breast cancer. Medical illness and positive life change: Can crisis lead to personal transformation?.

[B49] Guyatt G, Walter S, Norman G (1987). Measuring change over time: assessing the usefulness of evaluative instruments. J Chronic Dis.

[B50] Deyo RA, Diehr P, Patrick DL (1991). Reproducibility and responsiveness of health status measures. Statistics and strategies for evaluation. Control Clin Trials.

